# GWAS for plant growth stages and yield components in spring wheat (*Triticum aestivum* L.) harvested in three regions of Kazakhstan

**DOI:** 10.1186/s12870-017-1131-2

**Published:** 2017-11-14

**Authors:** Yerlan Turuspekov, Aida Baibulatova, Kanat Yermekbayev, Laura Tokhetova, Vladimir Chudinov, Grigoriy Sereda, Martin Ganal, Simon Griffiths, Saule Abugalieva

**Affiliations:** 1 Institute of Plant Biology and Biotechnology, Almaty, Kazakhstan 050040; 2 Kazakh Rice Research Institute, Kyzylorda, Kazakhstan 120016; 3 Karabalyk Breeding Station, Kostanai region, Kazakhstan 110908; 4 Karaganda Research Institute of Agriculture, Karaganda region, Kazakhstan 100435; 5 TraitGenetics Gmbh, 06466 Gatersleben, Germany; 6 John Innes Centre, Norwich Research Park, Norwich, NR47UH UK

## Abstract

**Background:**

Spring wheat is the largest agricultural crop grown in Kazakhstan with an annual sowing area of 12 million hectares in 2016. Annually, the country harvests around 15 million tons of high quality grain. Despite environmental stress factors it is predicted that the use of new technologies may lead to increases in productivity from current levels of 1.5 to up to 3 tons per hectare. One way of improving wheat productivity is by the application of new genomic oriented approaches in plant breeding projects. Genome wide association studies (GWAS) are emerging as powerful tools for the understanding of the inheritance of complex traits via utilization of high throughput genotyping technologies and phenotypic assessments of plant collections. In this study, phenotyping and genotyping data on 194 spring wheat accessions from Kazakhstan, Russia, Europe, and CIMMYT were assessed for the identification of marker-trait associations (MTA) of agronomic traits by using GWAS.

**Results:**

Field trials in Northern, Central and Southern regions of Kazakhstan using 194 spring wheat accessions revealed strong correlations of yield with booting date, plant height, biomass, number of spikes per plant, and number of kernels per spike. The accessions from Europe and CIMMYT showed high breeding potential for Southern and Central regions of the country in comparison with the performance of the local varieties. The GGE biplot method, using average yield per plant, suggested a clear separation of accessions into their three breeding origins in relationship to the three environments in which they were evaluated. The genetic variation in the three groups of accessions was further studied using 3245 polymorphic SNP (single nucleotide polymorphism) markers. The application of Principal Coordinate analysis clearly grouped the 194 accessions into three clades according to their breeding origins. GWAS on data from nine field trials allowed the identification of 114 MTAs for 12 different agronomic traits.

**Conclusions:**

Field evaluation of foreign germplasm revealed its poor yield performance in Northern Kazakhstan, which is the main wheat growing region in the country. However, it was found that EU and CIMMYT germplasm has high breeding potential to improve yield performance in Central and Southern regions. The use of Principal Coordinate analysis clearly separated the panel into three distinct groups according to their breeding origin. GWAS based on use of the TASSEL 5.0 package allowed the identification of 114 MTAs for twelve agronomic traits. The study identifies a network of key genes for improvement of yield productivity in wheat growing regions of Kazakhstan.

**Electronic supplementary material:**

The online version of this article (10.1186/s12870-017-1131-2) contains supplementary material, which is available to authorized users.

## Background

Hexaploid bread wheat (*Triticum aestivum* L.) is a major commodity for export in Kazakhstan and is grown annually on more than 12 million hectares. The history of wheat cultivation in Kazakhstan shows that most of wheat cultivars have been developed in collaboration with Russian breeders and using Russian wheat genetic resources [1]. Even after the breakup of the USSR, this trend is still in the place as the two countries share their expertise and genetic resources based on bilateral projects and international activities under the CIMMYT umbrella [2, 3]. It is also found that Kazakh-Russian wheat germplasm is genetically close to wheats from the USA [4]. It is hypothesized that the heavy importation of this crop from Russia at the end of the nineteenth century after the successful introduction of Turkish Red Wheat types to the US by Russian Mennonites [5] might be a main reason behind the close genetic relationship of Kazakh and US accessions [4].

Over 80% of the wheat harvesting area in Kazakhstan is grown with spring type and it is cultivated at higher latitudes in parts of the country, including Northern and North-Eastern Kazakhstan. The other important growing regions stretch along the Tian-Shan mountain chain in Southern and South-Eastern parts, where both winter and spring types are grown successfully. The climatic conditions in these regions are very variable, as are the soil types, the temperature during the growing season, the precipitation levels, and the photoperiod length [6]. Therefore, studies of yield performance in different ecological niches are important for strategies in current and future breeding activities across wheat growing regions of the country. It is projected that the improvement of agronomy and use of new breeding methods in this country may lead to the development of new varieties, and, consequently, improve the yield productivity up to 3 tons per hectares [7]. In the past, plant breeders successfully relied on using conventional tools and methodologies. Nowadays, the availability of new genomic tools and resources is leading to new opportunities to dissect the genetic mechanisms of complex traits associated with yield improvement [8].

As costs for high throughput genotyping are decreasing, genome-wide association studies (GWAS) are becoming a powerful approach for the detection of QTL (quantitative trait loci) associated with wheat agronomic traits, with the final goal of accelerating local breeding activities based on the application of marker-assistant selection [9, 10]. The success of GWAS in wheat is largely based on the development of high-density SNP genotyping platforms by Affymetrix [11, 12] and Illumina [9, 13], which are now providing rich resources for high-throughput genotyping data for wheat diversity panels. In GWAS, detection of significant associations relies primarily on genetic marker coverage, the number of individuals studied, and linkage disequilibrium (LD) between causative and linked polymorphisms [14, 15]. Currently, GWAS has been used successful in hexaploid wheat for identification of QTL for yield components [16–18], abiotic stress resistance [19–21] disease resistance [22, 23], and grain quality [24, 25]. A survey of the literature shows that GWAS is actively applied in wheat studies in many different parts of the World, including North America [26], Central America [25], Europe [18, 24], Africa [19], Australia [22], and Asia [20].

Although GWAS has proven to be a very efficient approach for capturing important marker-trait associations (MTA), results reported from studies in different regions of the World are revealing the tendency for a strong influence of the growth environment in which yield QTL are identified with significant genotype x environment interaction revealed (GEI). For instance, results obtained from three different GWAS studies related to identification of QTL for yield performance in Europe [24], India [27], and Mexico [16] showed different responses and QTL for yield components in different parts of the genome. This trend is also confirmed in studies when the same germplasm was tested in different regions of Asia [20]. This outcome is congruent with result reported by Quarrie et al. (2005) from studies using bi-parental mapping populations [28], and can be explained by the sensitivity to environmental factors at crucial growth phases which determines the potential number of grains per ear [29]. Therefore, the success of regional projects may largely depend on separate, local, GWAS experiments using genotyped adapted germplasm. The main goal of this work was GWAS using spring wheat accessions from Kazakhstan, Russia, Europe, and CIMMYT (Mexico) for identification of MTA in field trials in three diverse environments of Kazakhstan. The study is the first attempt to employ GWAS for identification of important QTL and enhancing of spring wheat breeding projects in this county.

## Methods

The spring wheat panel consisted of 96 commercial and prospective cultivars from Kazakhstan and the Russian Federation, 38 cultivars from Europe, and 60 CIMCOG (CIMMYT Mexico Core Germplasm) lines (CIMMYT, Mexico) (Additional file 1). Currently, 61 cultivars from Kazakhstan and Russia in this genetic panel have been registered through the State Seed Trials Commission of the Republic of Kazakhstan (2015), and are grown officially in Kazakhstan. The panel also included 29 prospective cultivars developed in Kazakhstan and Russia (Additional file 1). The European cultivar collection predominantly comprised accessions originating in the United Kingdom. The CIMCOG lines are a special population developed for studying opportunities for improvements in photosynthesis and biomass [30]. The field trials were conducted in three different latitude regions of Kazakhstan (Additional file 2), specifically at the Karabalyk breeding station (Northern Kazakhstan), Karaganda breeding Research Institute (Central Kazakhstan), and at the Kazakh Rice Research Institute (Southern Kazakhstan). The collection was planted at each site in randomized experiments each of three replicates in the seasons of 2013–2015. The distance between rows was 15 cm and the distance between plants within a row was 5 cm. The experiments in the Northern and Southern regions were conducted in 1 metre blocks, while in the Southern region the accessions were planted in 3 rows per repetition. In total, the data for mean values of 12 agronomic traits of the 194 hexaploid wheat accessions harvested in nine environments were subjected to further statistical analysis. The 12 traits included the following: days to booting (BD), days to heading time (HT), days to maturity (MT), thermal time at heading (TT-H), thermal time at maturity (TT-M), plant height (PH), peduncle length (PL), number of fertile spikes (NFS), number of kernels per spike (NKS), thousand kernel weight (TKW), dry biomass per plant (BPP) and yield per plant (YPP).

DNA samples were extracted and purified from single seeds of individual cultivars using commercial kits (Qiagene, CA, USA). The DNA concentration for each sample was adjusted to 50 ng/μl. Accessions were genotyped using the wheat 90 K Illumina iSelect SNP array as described in [4].

Statistical analyses of data, including multiple factor ANOVA, Pearson’s correlation and *t-test* were calculated using the software package GraphPad Prism 5.0 [31]. GGE Biplot methods were employed by using the GenStat package (17th release, VSN International, Hertfordshire, UK). The symmetric scaling option of both methods and available field data for all three sites were used in estimations.

GWAS analysis of QTL governing plant growth stages and yield parameters in the set of 194 accessions was performed with the TASSEL 5.0 package [32]. For this, the SNP dataset was filtered using a 10% cutoff for missing data and only markers with a minor allele frequency ≥ 0.10 were considered for GWAS. The STRUCTURE and STRUCTURE HARVESTER [29] programs were used for the development of delta K values (ΔK) and Q-matrix for identified clusters.

## Results

### Field performance of the spring wheat collection in three regions of Kazakhstan

**Fig. 1:**
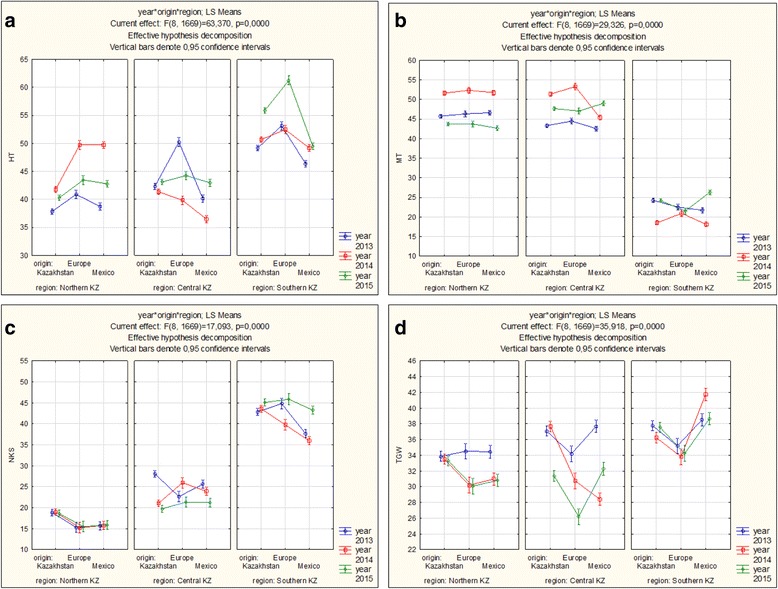
Average HT (**a**), MT (**b**), NKS (**c**) and TKW (**d**) of 194 wheat accessions of three breeding origins harvested in the three regions of Kazakhstan during 2013–2015. *Bars* denotes 95% confidence interval

Data on field performance of the 194 spring wheat accessions from Europe (EU), CIMMYT (CIMCOG lines), and lines from Kazakhstan and the Russian Federation (Additional file 1) were analyzed at the experimental stations of Northern, Central and Southern regions of Kazakhstan during the 2013–2015 seasons. The length of the plant growth phases, and means of yield components in the collection from the three breeding origins were significantly different among the regions (Fig. 1). While heading time of samples from all three regions were earlier in Northern and Central regions in comparison to South Kazakhstan (Fig. 1a), maturation time length was exactly opposite (Fig.1b). Yield components, including the number of kernels per spike (NKS), was always higher in the Southern breeding station (Fig. 1c). Average YPP (2013–2015) for the three groups was not correlated across the three regions, except CIMCOG lines in the Southern region, which were significantly correlated with local and EU cultivars in the Central region (Additional file 3). In Northern Kazakhstan, which is the most important wheat growing region of the country, the yield was lowest of the sites during all the years studied. The Pearson’s correlation test suggested that leading contributing factors to the YPP in Northern Kazakhstan were BD, PH, BPP, NFS, and NKS (Additional file 4). In total, 10 different observations were measured for plant growth phases in the North, Center, and South of Kazakhstan, including TT-H and TT-M dates. The Pearson correlation index suggested that in all three regions TT-H and TT-M exerted a highly significant influence on yield components, including the number of kernels per spike (NKS) and thousand grain weight (TKW) (Additional file 4).

The two developmental phases of growth, TT-H and TT-M, in this study differed among breeding origins (O effect in Table 1 and Fig. 1), and showed a significant interaction with breeding origin within the nine environments (O x R x Y) and places of growth (O x R).

**Table 1:** Three-way ANOVA performed on the traits studied in nine environments

Source	d.f.	TT-H	TT-M	PH	NKS	TKW	Yield/p
Year	2	1570.4***	3153***	313.2***	12.77***	135.5***	54.8***
Origin	2	384.9***	312***	1257.9***	62.27***	130.8***	156.4***
Region	2	6394.2***	5395***	722.7***	5610.34***	336.1***	1908.5***
Year x Origin	4	31.8***	18***	36.5***	7.07***	15.8***	28.9***
Year x Region	4	946.1***	2230***	129.9***	56.75***	41.7***	195.2***
Origin x Region	4	123.3***	126***	88.8***	38.67***	48.1***	56.3***
Year x Origin x Region	8	28.8***	24***	69***	17.09***	35.9***	62.3***

The F-values are provided with significance level indicated by the asterisks

***P < 0.001

The magnitude of the main effects for yield (Year, Region, and Origin), and their interactions were ranked region > origin > year > Y x R > Y x O x R > O x R, and the least effect was Y x O, as indicated by the F-values (Table 1). The only case where the origin effect was greater over the regional effect was the result received from the PH, where the F-value for the origin effect was 1.7 times higher than for the region effect.

**Fig. 2:**
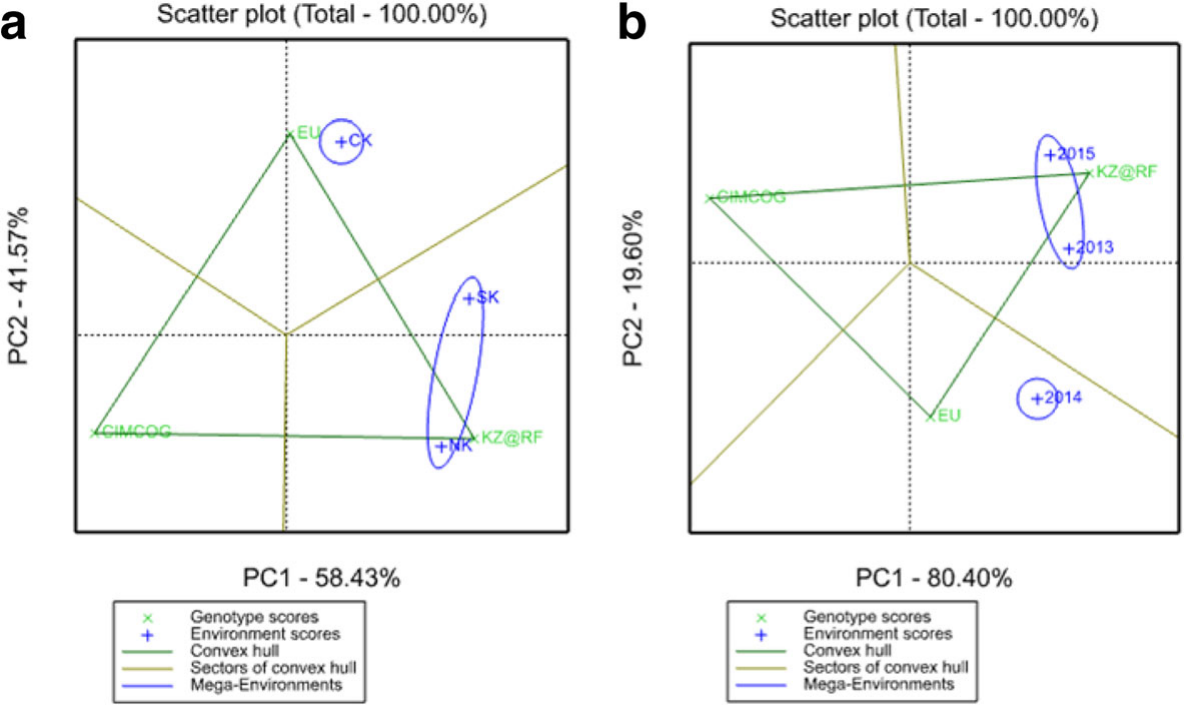
GGE biplot scans for the regional effect (**a**) and the year effect (**b**) using the yield performance of wheat accessions from three breeding origins studied in nine environments of Kazakhstan. *Green points* indicate the breeding origins, *blue color* represents the region (**a**) and the year (**a**) effects

The GGE biplot analysis, based on yield performance in the nine environments, is separated into groups with different breeding origins both by region and year effects (Fig. 2). In the analysis of the regional effect the biplot indicates that the South and North of Kazakhstan are more suitable for accessions from Kazakhstan and the Russian Federation, while Central Kazakhstan is more favorable for accessions from Europe (Fig. 2a). In the analysis of the year effect, the group of wheat accessions from Kazakhstan and the Russian Federation was well matched to the environments of 2013 and 2015, while in 2014 was more favorable for wheat accessions from Europe (Fig. 2b). Although the three groups of accessions with different breeding origins were well separated in both scans, the separation of environment effects was different. In the case of the regional effect, both principal coordinates were efficiently discriminating the 2 mega-environments, but in the case of the year effect the separation of the 2 mega-environments was largely based on the second principal coordinate (PC2).

### Genetic variation of spring wheat with different breeding origins based on SNP data

The 194 spring wheat accessions from Kazakhstan, Russia, and Western Europe were genotyped using the 90 K SNP iSelect array containing 81,587 SNPs. The genotyping allowed the generation of 66,223 scorable SNPs and *Blastn* search allowed the identification of 55,165 SNP hits in the chromosome survey sequencing project of IWGSC [33] allowing the chromosomal location of the markers. The genotyping data for CIMCOG lines was freely available at [34] and generated by using the 35 K Axiom® array [12] [32]. From these data, the genetic variability amongst the 194 spring wheat accessions, including the 60 CIMCOG lines, was studied based on 3245 polymorphic SNP markers. The number of polymorphic markers was reduced after alignment of the three groups of accessions, whereby the smallest datasets were for the CIMCOG group (3852 SNP markers). The total length of all wheat chromosomes in the SNP map was 3109.9 cM. The average SNP density was 0.96 SNP/cM and ranged from 0.68 in the B genome to 2.31 in the D genome. The range of Nei’s unbiased diversity index in the three groups of accessions varied from 0,247 in the CIMCOG lines to 0,339 in the mixed group of Kazakhstan and Russian accessions (Table 2). The diversity index for the genomes A, B, and D were 0.286, 0.284, and 0.269, respectively. The D genome had the largest LD (*r* ^2^ 0.1) blocks (26.8 cM) followed by the A genome (17.5 cM) and the B genome (14.0 cM) genome (Additional file 5).

**Table 2:** Genetic diversity indices in the three groups of spring wheat accessions using 3245 SNPs analyzed using GeneAlex

Accession groups	N	Ne	I	Uh
Kazakhstan-Russia	96	1.563 + 0.005	0.507 + 0.003	0.339 + 0.002
West Europe	38	1.428 + 0,006	0.409 + 0.004	0.270 + 0.003
CIMCOG	60	1.393 + 0,006	0.379 + 0.004	0.247 + 0.003

*N* number of accessions, *Ne* number of effective alleles, *I* Shannon Information index, *Uh* Unbiased Nei’s Diversity index

**Fig. 3:**
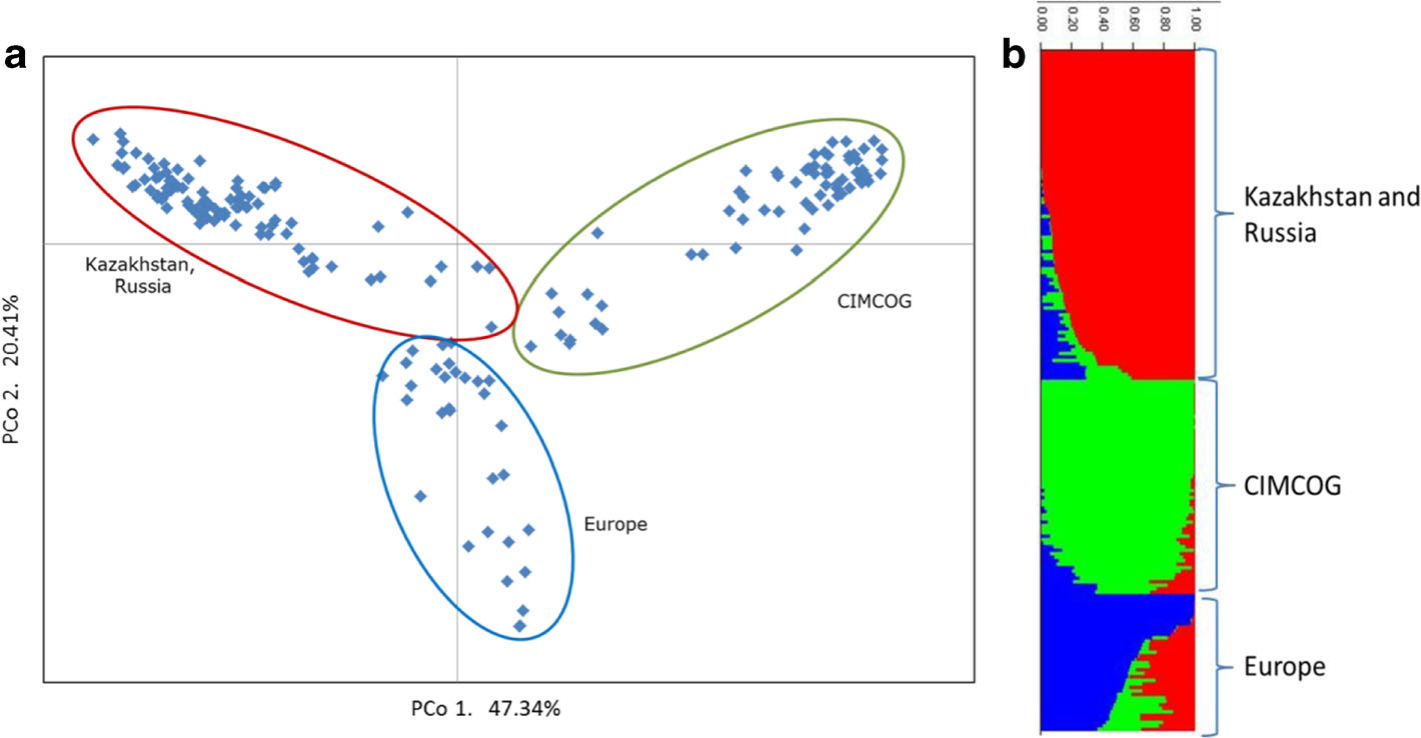
Genetic differentiation of 194 spring wheat accessions using 3245 SNP markers. **a**. Principal Coordinate analysis of wheat with the three breeding origins clustered using GenAlEx version 6.5. **b**. Clustering of samples using the STRUCTURE software

The Principal Coordinate analysis revealed the separation of the accessions into the three distinct subgroups according to their breeding origin (Fig. 3). The first coordinate (47.34%) clearly separated Kazakhstan and Russia samples from CIMCOG lines, and the second coordinate (20.41%) separated European accessions from the other two groups (Fig. 3a). This result was congruent with the outcome from the STRUCTURE analysis where the accessions in the three clades were separated according to their breeding origin (Fig. 3b).

### Identification of SNP markers for growth stages and yield components based on GWAS

In this study, the genotyping HapMap file consisted of 194 spring wheat accessions and 3245 polymorphic SNP markers. The set of polymorphic SNP data was prepared after filtering with a 10% of cutoff for missing data and markers with minor allele frequencies ≥ 0.10. After running Structure Harvester, a Q-matrix for the three identified clusters was selected for further analysis based on analysis of delta K value (ΔK). The genotyping set was analyzed separately for each of nine studied environments in Northern, Central, and Southern Kazakhstan.

**Fig. 4:**
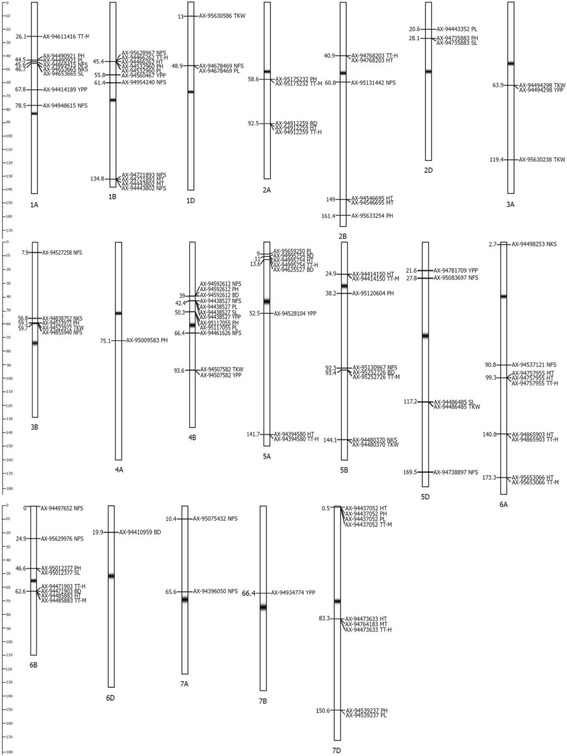
Chromosomal locations of SNP markers associated with agronomic traits in common wheat. SNP and trait names given on *right side* of the chromosomes. Positions of SNPs shown in cM on *left side* of chromosomes

In total, 114 MTAs were identified on 19 chromosomes of the wheat genome (Fig. 4., Additional file 6) and the largest number of associated markers was detected on chromosome 4B (12 MTAs). Manhattan and QQ (quintile - quintile) plots of identified MTAs in the North, Center and South regions are given in Additional files 7, 8, and 9, respectively. Forty six MTAs were identified for traits related to length of the growing stages, 68 MTAs were identified for morphological traits and yield components, and six markers showed significance for both groups of traits (Additional file 6). Twelve QTL were simultaneously identified in two environmental sites within the region. Only two MTAs for PL mapped on the 1B and 7D chromosomes (AX-94532960 and AX-94539237) were significant for environmental sites among regions. Among MTAs for agronomic traits, 21 associations were identified for NFS, four for NKS, and seven for TGW (Additional file 6).

## Discussion

To facilitate the discovery of MTAs, three groups of wheat accessions with different breeding origins were studied. In genetic terms, the accessions were clearly separated into the three subgroups, with the first subgroup (Russia/Kazakhstan) being the most diverse, and the third subgroup (CIMCOG) being the least diverse group in the analysis. The separation of accessions into the three subgroups was also congruent with population structure analysis using on STRUCTURE software.

Yield performance during the 3 years was lowest in Northern Kazakhstan. Yield performance in the Southern region was always higher. However, the size of arable land in this region is limited and insignificant in comparison with the wheat growing area in Northern Kazakhstan. Results suggest that accessions in the EU collection can successfully be used in the Central region for obtaining better yield, and selected CIMCOG lines can be efficiently used for improvement of TKW in all three regions. The GGE biplot based on yield data helps to confirm that groups within the three breeding origins have different genetic backgrounds, and local accessions are well adapted to Northern and Southern regions.

Incorporation of the CIMCOG lines in the analysis resulted in a reduction of polymorphic SNP markers available for GWAS, as genotyping data for those lines was restricted to those shared between the Axiom and Illumina SNP arrays. Therefore, only 3245 aligned polymorphic SNPs were used in the GWAS with 1340 SNPs positioned in the A genome, 1448 in the B genome, and 457 in the D genome. The Diversity index was relatively high in the A and B genomes and lower in the D genome, which is well in agreement with previous observations [10, 18]. GWAS was performed separately for nine field trials over 3 years and identified 114 MTAs (Additional file 6). Only 12 of the identified MTA were significant in two environments both within and between regions (Additional file 6), suggesting that stability of the associations was undermined by a strong influence of environmental factors.

A comparison of the MTAs detected in this study with those reported in other publications indicates a number of similarities. For instance, the *Rht-D1* gene is known to be located at 31.5 cM on chromosome 4B [35] and close to AX-94592612 (39.0 cM), which in this study is associated with plant height, days to booting and number of fertile spikes. Other example of similarities are the locations of MTAs for peduncle length (20.6 cM), plant height and spike length (both at 28.1 cM) (Table 3), and the position of the *Rht8* gene on chromosome 2D (23.0 cM) [36]. A MTA for NKS on chromosome 6A (2.7 cM) in this study was positioned at a similar location as in the study by Guo et al. [24]. MTAs for PH on chromosome 4B (AX-94592612 and AX-95117055) have a similar position to the *Rht-B1* gene for plant height identified using a bi-parental mapping population tested in South-east of Kazakhstan [28]. In the same study by Quarrie et al. [28], QTL for NKS (chromosomes 1A), TKW (1D, 3A, and 4B), and NFS (1A, 4B, 5D, and 7A) were detected in similar positions for MTAs of NKS, TKW and NFS found in this study (Table 3).

**Table 3:** List of SNPs for selected yield components identified in this study and comparison of their locations to QTL mapped elsewhere

N	Trait	SNP	Chr	Position (cM)	Sukumuran et al. (2015)	Zanke et al. (2015)	Jaiswal et al. (2016)	Guo et al. (2017)	Quarrie et al. (2005), ranges in cM
1	NFS	AX-94869415	1A	45.65					39.5–47.5
2	NFS	AX-94948615	1A	78.56					
3	NFS	AX-95628967	1B	45.57					
4	NFS	AX-94954240	1B	61.49					
5	NFS	AX-94443802	1B	134.89					
6	NFS	AX-94678469	1D	48.90					
7	NFS	AX-95131442	2B	60.89					
8	NFS	AX-94527258	3B	7.98					
9	NFS	AX-94855940	3B	59.75					
10	NFS	AX-94592612	4B	39.00					29.7–91.2
11	NFS	AX-94461626	4B	66.40					29.7–91.2
12	NFS	AX-95130967	5B	92.30					
13	NFS	AX-95083697	5D	27.86					
14	NFS	AX-94738897	5D	169.57					154.6–175.6
15	NFS	AX-94537121	6A	90.86					
16	NFS	AX-94497652	6B	0.00					
17	NFS	AX-95629976	6B	24.90					
18	NFS	AX-95075432	7A	10.48					0–22.9
19	NFS	AX-94396050	7A	65.60					
20	NKS	AX-94653665	1A	46.79			47.0		39.5–47.5
21	NKS	AX-94838752	3B	56.89					
22	NKS	AX-94480370	5B	144.10					
23	NKS	AX-94498253	6A	2.72				5.7	
24	PH	AX-94490921	1A	44.51					
25	PH	AX-94532960	1B	45.57					
26	PH	AX-95175232	2A	58.66					
27	PH	AX-95633254	2B	161.40					
28	PH	AX-94735883	2D	28.18					
29	PH	AX-94523972	3B	59.17					
30	PH	AX-95009583	4A	75.10					
31	PH	AX-94592612	4B	39.00					
32	PH	AX-95117055	4B	50.38					
33	PH	AX-95120604	5B	38.20					
34	PH	AX-95012377	6B	46.69			42.0		
35	PH	AX-94437052	7D	0.57					
36	PH	AX-94539237	7D	150.63					
37	PL	AX-94490921	1A	44.51					
38	PL	AX-94532960	1B	45.57					
39	PL	AX-94678469	1D	48.90			49.0		
40	PL	AX-94443352	2D	20.69					
41	PL	AX-95117055	4B	50.38					
42	PL	AX-95659250	5A	9.09					
43	PL	AX-94437052	7D	0.57					
44	PL	AX-94539237	7D	150.63					
45	SL	AX-94653665	1A	46.79			47.0		
46	TKW	AX-95630586	1D	11.02		12.1			7.5–29.0
47	TKW	AX-94494298	3A	63.91					
48	TKW	AX-95630238	3A	119.14					97.7–152.8
49	TKW	AX-94523972	3B	59.17	61.0				
50	TKW	AX-94507582	4B	93.60					94.2–139.2
51	TKW	AX-94480370	5B	144.10					
52	TKW	AX-94486485	5D	117.24					

Despite similarities in the genetic positions of MTAs identified with other studies, a number of MTAs from this study were missing in GWAS conducted in different geographic regions. This study revealed four MTAs related to NKS, which is one of the major yield components in wheat [28]. Surveys of the literature suggests that the loci identified on chromosomes 3B (56.9 cM) and 5B (144.1 cM) were not identified in previous GWAS studies in other regions of the World [17, 25, 28] nor in Quarrie et al. [28]. Therefore, it was hypothesized that these MTAs, along with other associations shown in Table 3, are new MTAs.

Recently, there have been a number of discussions related to the importance of size and level of genetic variation in diversity panels for the success of GWAS projects [14, 15, 37]. It was pointed out that experiments with less than 384 accessions [14] and large LD blocks [15] may lead to the identification of false positive associations. The study by Turner et al. (2016) indicated that smaller panels may allow the detection of false negative associations that would not have been detected in the larger panels [37]. Results in this study using a relatively small panel (*n* = 194) are largely in agreement with the study by Turner et al. [37].

## Conclusion

The study confirms the efficiency of GWAS for the identification of molecular markers which tag important agronomic traits. In total 114 MTAs for 12 physiological and agronomic traits determined using spring wheat samples from Kazakhstan, Russia, EU and CIMMYT studied in field conditions of three regions of Kazakhstan. Locations of identified MTAs for plant height were similar with genetic positions corresponding to *Rht-B1, Rht-D1,* and *Rht8* genes of wheat. In addition, from field trials it was found that EU and CIMMYT germplasm has high breeding potential to improve yield performance in Central and Southern regions of the country. The use of Principal Coordinate analysis clearly separated the panel into three distinct groups according to their breeding origin. The study identifies a network of key genes that will be further validated for improvement of yield productivity in wheat growing regions of Kazakhstan.

## Additional files

Additional file 1: List of accessions from Kazakhstan, Russia, Europe, and Mexico analyzed in the study. (XLS 43 kb)

Additional file 2: Location and climate data of three breeding sites in Kazakhstan. (DOC 44 kb)

Additional file 3: Correlation of average YPP over three years among experimental sites in relationship to breeding origin of accessions. (XLS 27 kb)

Additional file 4: Pearson correlation analysis between traits in Northern Kazakhstan during 2013–2015. (XLS 70 kb)

Additional file 5: LD decay lines (threshold *r* ^2^ 0.1) for the A, B, D genomes, and whole genome based on 3245 SNP markers. A. A genome; B. B Genome; C. D genome; D. whole genome. (DOC 294 kb)

Additional file 6: The list of MTAs identified based on 3245 SNP markers using TASSEL 5.0. (XLS 61 kb)

Additional file 7: Manhattan and QQ plots based on the analysis of field plot data from North Kazakhstan analyzed using the TASSEL 5.0 package. (XLS 6271 kb)

Additional file 8: Manhattan and QQ plots based on the analysis of field plot data from Center Kazakhstan using the TASSEL 5.0 package. (XLS 11565 kb)

Additional file 9: Manhattan and QQ plots based on the analysis of field plot data from South Kazakhstan using the TASSEL 5.0 package. (XLS 10628 kb)
