# Supplementary material for: GWAS for plant growth stages and yield components in spring wheat (Triticum aestivum L.) harvested in three regions of Kazakhstan
Source: BMC Plant Biol. 2017 Nov 14;17(Suppl 1):190. doi: 10.1186/s12870-017-1131-2 (PMC5688510; doi:10.1186/s12870-017-1131-2)
Supplement: Supplementary file 2 — Location and climate data of three breeding sites in Kazakhstan. (DOC 44 kb) [file 12870_2017_1131_MOESM2_ESM.doc]

Additional File 2. Location and climate data of three breeding sites in Kazakhstan

| **Index** | Karabalyk,  Northern Kazakhstan | | | Karaganda,  Central Kazakhstan | | | Kyzylorda,  Southern Kazakhstan | | |
| --- | --- | --- | --- | --- | --- | --- | --- | --- | --- |
|  | 2013 | 2014 | 2015 | 2013 | 2014 | 2015 | 2013 | 2014 | 2015 |
| Average rainfall, mm | 294 | | | 110 | | | 45 | | |
| Annual rainfall, mm | 425.2 | 301.7 | 155.8 | 103.7 | 109.5 | 116.9 | 36.2 | 30.0 | 67.3 |
| Veg. period rainfall | 10.7 | 109.5 | 46.2 | 12.2 | 26.4 | 65.2 | 22.6 | 18.9 | 27.9 |
| Mean T⁰C | 18.1 | 19.8 | 19.9 | 11.3 | 18.3 | 18.5 | 22.1 | 22.4 | 20.6 |
| Max T⁰C | 36.0 | 36.0 | 44.0 | 25.3 | 37.0 | 38.8 | 39.5 | 42.8 | 44.6 |
| Min T⁰C | 0.0 | 5.0 | 4.0 | 12 | -0.5 | -0.5 | 2.0 | -2.5 | 0.4 |
| Veg. period mean T⁰C | 18.1 | 19.5 | 19.9 | 9.4 | 18.8 | 19.2 | 17.2 | 14.5 | 16.6 |
| Soil type | Black soil  (4.5-5% humus) | | | Dark chesnut  (humus 3.0-3.5%) | | | Meadow-marsh | | |
| Latitude | 53.45 | | | 49.4 | | | 44.51 | | |
| Longitude | 62.03 | | | 72.41 | | | 65.30 | | |
| Elevation above sea level | 189 | | | 570 | | | 129 | | |
| Date of sowing | 14 May | 15 May | 16 May | 26 May | 24 May | 1  June | 4  Apr | 3  Apr | 5  Apr |
| Date of harvesting | 17 Aug | 29 Aug | 24 Aug | 3 Sept | 1 Sept | 10 Sept | 12  July | 1 July | 10  July |
| Irrigation* | R | R | R | R | R | R | I | I | I |

*R – Rainfed, I – Irrigated.
