# Supplementary material for: GWAS for plant growth stages and yield components in spring wheat (Triticum aestivum L.) harvested in three regions of Kazakhstan
Source: BMC Plant Biol. 2017 Nov 14;17(Suppl 1):190. doi: 10.1186/s12870-017-1131-2 (PMC5688510; doi:10.1186/s12870-017-1131-2)

Additional File 5. LD decay lines (threshold *r*^2^ 0.1) for the A, B, D genomes, and whole genome based on 3245 SNP markers. A. A genome; B. B Genome; C. D genome; D. whole genome.

A B


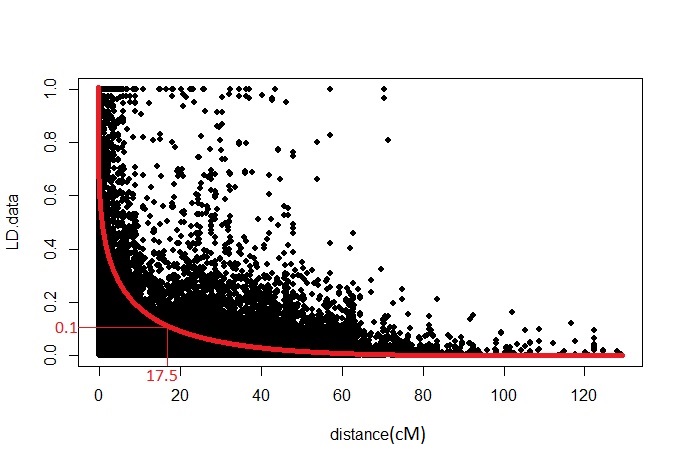

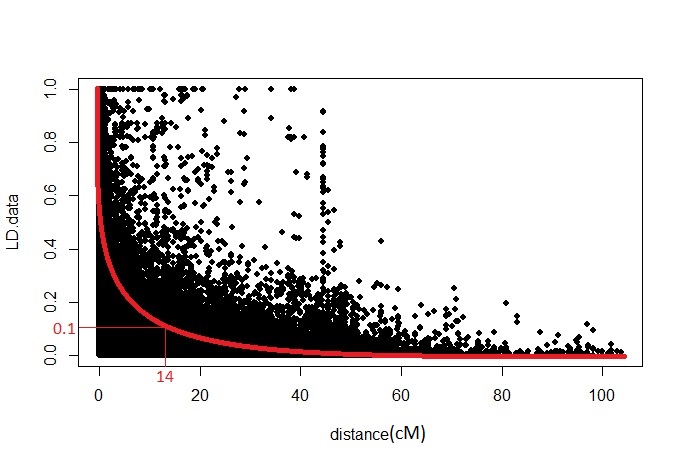


C D


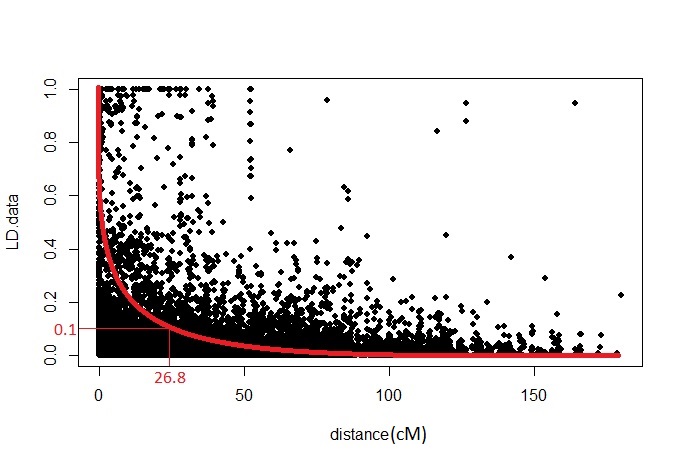

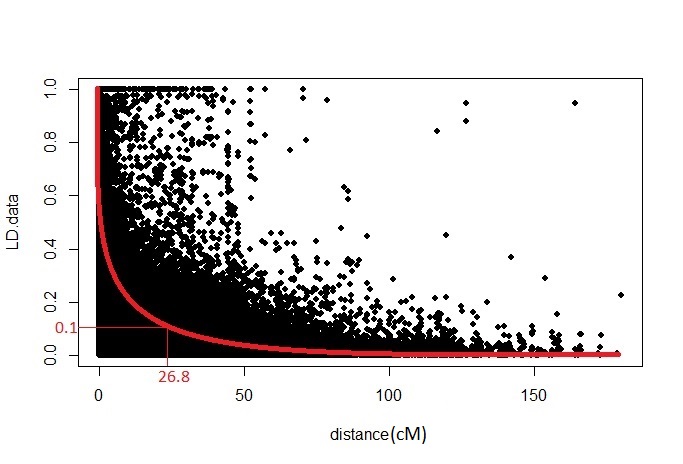

Supplement: Supplementary file 5 — LD decay lines (threshold r 2 0.1) for the A, B, D genomes, and whole genome based on 3245 SNP markers. A. A genome; B. B Genome; C. D genome; D. whole genome. (DOC 294 kb) [file 12870_2017_1131_MOESM5_ESM.doc]
